# Effectiveness of Horticultural Therapy in People with Schizophrenia: A Systematic Review and Meta-Analysis

**DOI:** 10.3390/ijerph18030964

**Published:** 2021-01-22

**Authors:** Shan Lu, Yajie Zhao, Jianjiao Liu, Feng Xu, Zhiwen Wang

**Affiliations:** 1 College of Horticulture, China Agricultural University, Beijing 100069, China; ls2502@163.com (S.L.); liujianjiao@hotmail.com (J.L.); 2 School of Nursing, Peking University, Beijing 100069, China; zhaoyajie@bjmu.edu.cn

**Keywords:** horticultural therapy, schizophrenia, meta-analysis, systematic review

## Abstract

Horticultural therapy is increasingly being used in the non-pharmacological treatment of patients with schizophrenia, with previous studies demonstrating its therapeutic effects. The healing outcomes are positively correlated with the settings of the intervention. This review aimed to evaluate the effectiveness of horticultural therapy on the symptoms, rehabilitation outcomes, quality of life, and social functioning in people with schizophrenia, and the different effectiveness in hospital and non-hospital environments. This review followed the Preferred Reporting Items for Systematic Review and Meta-Analysis Protocols (PRISMA) guidelines. We researched studies through PubMed, Embase, the Cochrane Library, Science Direct, and the China National Knowledge Infrastructure. We included randomized controlled trials (RCTs) and quasi-experimental studies about horticultural therapy for people with schizophrenia, from January 2000 to December 2020, with a total of 23 studies involving 2024 people with schizophrenia included in this systematic review. This study provided evidence supporting the positive effect of horticultural therapy. This review demonstrated that non-hospital environments have a better therapeutic effect on all indicators than hospital environments. The results also demonstrated the effectiveness of horticultural therapy on symptoms, rehabilitation outcomes, quality of life, and social functioning in patients in hospital and non-hospital environments, providing further evidence-based support for landscape design.

## 1. Introduction

Schizophrenia is one of the most common severe mental disorders, being ranked among the top 20 causes of disability worldwide [1] and affecting 20 million people [2]. People with schizophrenia often share common experiences, such as hallucinations, delusions, disturbances of emotions, and distortions in behavior and language, and they face 2–3 times the risk of early death than the general population [3], qualifying the severity of this mental disorder. Schizophrenia is a debilitating disease because patients are cognitively impaired, which is often related to decreased executive functioning, eventually leading to severely impaired daily functioning and social interactions. 

As schizophrenia is a chronic relapsing disease with a high recurrence rate and a high possibility of disability, the treatment of it has become one of the most challenging issues, affecting not only the everyday life of patients but also their family financial status [4]. Currently, medication is the primary treatment for schizophrenia. However, the long-term usage of antipsychotic drugs poses some risks, such as metabolic syndrome, manifested in weight gain and diabetes [5]. Recent evidence has demonstrated that non-pharmacological therapies are more desirable to alleviate symptoms of schizophrenia without producing side effects [6,7]. Horticultural therapy has received increasing attention as an effective and non-pharmacological intervention [8]. Horticultural therapy is defined by the American Horticultural Therapy Association (AHTA) as the engagement of a person in gardening-related activities, facilitated by a trained therapist, to achieve specific treatment goals. It can be used as a useful tool in physical and emotional treatment [9].

In the past two decades, increasing numbers of studies have focused on the effectiveness of horticultural therapy on people with schizophrenia, many of which have shown that it can alleviate patients’ negative and positive symptoms and the severity of mental disability [10,11,12,13,14,15,16]. The treatment can also have physical benefits [12,13,17] and improve social functioning [14,17,18,19]. As previously mentioned, horticultural therapy could result in better therapeutic outcomes than standard care for schizophrenia. However, the effectiveness of horticultural therapy is still unclear. From this perspective, the magnitude of these differences must be quantitatively analyzed by conducting systematic reviews and meta-analyses. A meta-analysis is a quantitative and comprehensive evaluation of the results of several studies [20], being useful for mitigating some problems such as small sample sizes and low statistical power [21]. Therefore, the results of meta-analyses are more precise and can yield more accurate outcomes for horticultural therapy interventions for patients with schizophrenia.

However, only one meta-analysis [22] has been conducted (in 2014) to evaluate the effects of horticultural therapy for people with schizophrenia or schizophrenia-like illnesses, which compared horticultural therapy with conventional workshop training, and only one study was included [17]. It mentioned the lack of clear evidence of the differences between pre- and post-measurement data on quality of life and wellbeing, but a combination of horticultural therapy and standard care might be more effective than routine maintenance in relieving symptoms of depression, stress, and anxiety in the short term according to that review. Therefore, more research is needed to provide adequate support for the effectiveness of horticultural therapy. For the purpose of this study, we evaluated the effects of horticultural therapy on schizophrenia through a systematic review (i.e., symptoms, rehabilitation outcomes, quality of life, and social functioning).

Meta-analyses can also be used to draw new conclusions from previous studies by investigating the different impacts of different conditions and dividing the studies into subgroups. Previous studies with different research foci can be further divided into several subgroups according to the type of activity (participatory horticultural therapy and ornamental horticultural activities [23]; horticultural intervention and community gardening [24]; raising plants, plant decoration, and combination activities [25]), participants (gardeners and non-gardeners [24,26] and different age groups [25]), country (U.S., U.K., and Asia [24]), respondents (patients or non-patients [26]), gardening (therapy vs. non-therapy [26]), subject types (child, teenager, adult, or elderly [26]), etc. Overall, these studies focused on the characteristics of activities and populations. 

In the last several years, a growing body of studies that differ in intervention settings has explored the effectiveness of horticultural therapy. There has been no meta-analysis published on the use of horticultural therapy as a treatment option for schizophrenia in different program settings. Therefore, a meta-analysis providing evidence for the link between effectiveness and program settings is necessary. Various studies have confirmed that the environment significantly contributes to improving patient conditions in the process of rehabilitation [27,28,29,30,31,32]. The non-hospital settings in some included studies are more similar to nature. Based on the above, we hypothesized that therapeutic outcomes are related to different environments, and that non-hospital environments would be more effective. Therefore, from the perspective of landscape researchers, we highlight the different influences of the settings (non-hospital vs. hospital environments) of horticultural therapy in the subgroup analysis.

## 2. Materials and Methods

In this review, we followed the Preferred Reporting Items for Systematic Review and Meta-Analysis Protocols (PRISMA) guidelines [33].

### 2.1. Search Strategy

Studies from January 2000 to December 2020 were searched and collected in this study. We searched PubMed, the Cochrane Library, Embase, and Science Direct using common keywords: (horticul* OR floricult* OR arboricult* OR olericult* OR agricult* OR garden* OR farm*) AND schizophrenia. The search strategy for the China National Knowledge Infrastructure was as follows: “(SU = ‘花园(garden)’ OR SU = ‘园艺(horticulture)’ OR SU = ‘农(farm)’) AND SU = ‘精神分裂症(schizophrenia)’”.

### 2.2. Inclusion and Exclusion Criteria

A description of the inclusion/exclusion criteria is provided in Table 1, according to the population, intervention, comparison, outcomes, and study design (PICOS).

The measuring tools of the symptoms included the Positive and Negative Syndrome Scale (PANSS), the Brief Psychiatric Rating Scale (BPRS), and the Scale for Assessment of Negative Symptoms (SANS). The Inpatient Psychiatric Rehabilitation Outcome Scale (IPROS) was used to measure rehabilitation outcomes. The Schizophrenia Quality of Life Scale (SQLS) and the Generic Quality of Life Inventory-74 (GQLI-74) were used to explore the quality of life, while the measuring tools of social functioning included the Scale of Social Function in Psychosis Inpatients (SSPI), Personal and Social Performance (PSP) scale, and the Social Disability Screening Schedule (SDSS).

### 2.3. Selection of Articles

We imported all studies into EndNote X7. Duplicate studies were excluded, and then we screened the studies by the titles, abstracts, and full texts according to the inclusion and exclusion criteria of this review. If two independent reviewers disagreed, it was resolved through discussion or by a third reviewer.

### 2.4. Quality Evaluation

Two independent reviewers critically appraised the quality of the eligible studies. For the RCTs, we evaluated the risk of bias for the included literature using the RCT-specific bias risk assessment tool in the Cochrane handbook for systematic reviews of interventions [34], which assesses randomization procedure biases, allocation concealment, and selective reporting. We used the Joanna Briggs Institute (JBI) critical appraisal tools for the quasi-experimental studies [35].

### 2.5. Statistical Methods

#### 2.5.1. Data Extraction

In terms of data extraction, we read the title and abstract. After excluding irrelevant documents, we read the full text to determine whether it should be included and then summarized the information. The data extraction mainly included: (1) basic information, including research title, first author, and publication time; (2) baseline characteristics of the research subjects, including the number, age, and sex of people included in each group and the disease diagnosis criteria of the study subjects; (3) specific details of intervention measures, including intervention form, time, and settings; (4) critical elements of bias risk assessment; (5) the outcome indicators and outcome measurement data concerned.

#### 2.5.2. Statistical Analysis

We pooled the data of the individual studies using Revman5.3 software. A random effects model was used, assuming heterogeneity between the studies and their respective effect sizes. We used standardized mean differences (SMDs) and mean differences (MDs). The results were aggregated with 95% confidence intervals (CIs). A *p*-value < 0.05 was considered statistically significant. The standard I^2^ tests were used to assess the statistical heterogeneity and we conducted a sensitivity analysis to evaluate the reliability and stability of the results.

We used subgroup analysis to investigate the effects of the different intervention environments (non-hospital vs. hospital environments) on the symptoms, rehabilitation outcomes, quality of life, and social functioning of patients.

## 3. Results

### 3.1. Search Outcomes

**Figure 1:**
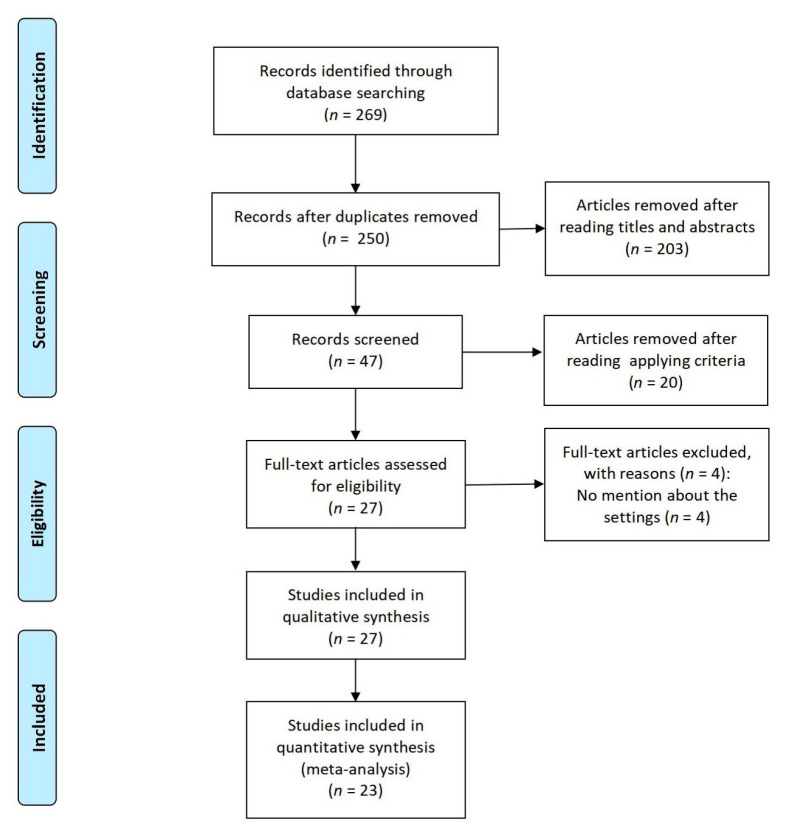
Flow diagram for the systematic review process.

Figure 1 explains our review process. We found 269 articles from PubMed (*n* = 139), the Cochrane Library (*n* = 1), Embase (*n* = 5), Science Direct (*n* = 73), and the China National Knowledge Infrastructure (*n* = 51). We removed 19 articles because of duplication, as well as 203 after reading the titles and abstracts. Of the remaining 47 articles, 12 were removed because they did not meet the inclusion criteria, five because of a lack of a control group in the intervention programs, two because they were reviews, and one because it did not have a baseline assessment. As this review was divided into two subgroups according to the settings, four other articles without mention of this information were excluded. Finally, 23 studies were included (Figure 1).

### 3.2. Study Characteristics

The features of the selected studies are aggregated. The number of people ranged from 28 to 615 (2024 in total) and their ages ranged from 15 to 65 years. Most horticultural therapy activities included growing flowers or vegetables, daily maintenance, and doing handicrafts. The settings were hospitals, agricultural rehabilitation training institutions, farms, and communities, which we divided into hospital and non-hospital environments.

### 3.3. Methodological Quality

**Figure 2:**
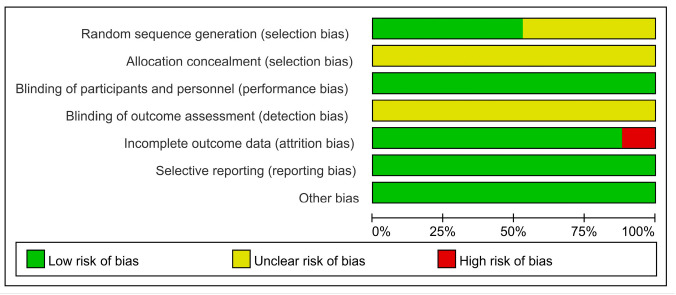
Risk of bias graph of included studies.

**Figure 3:**
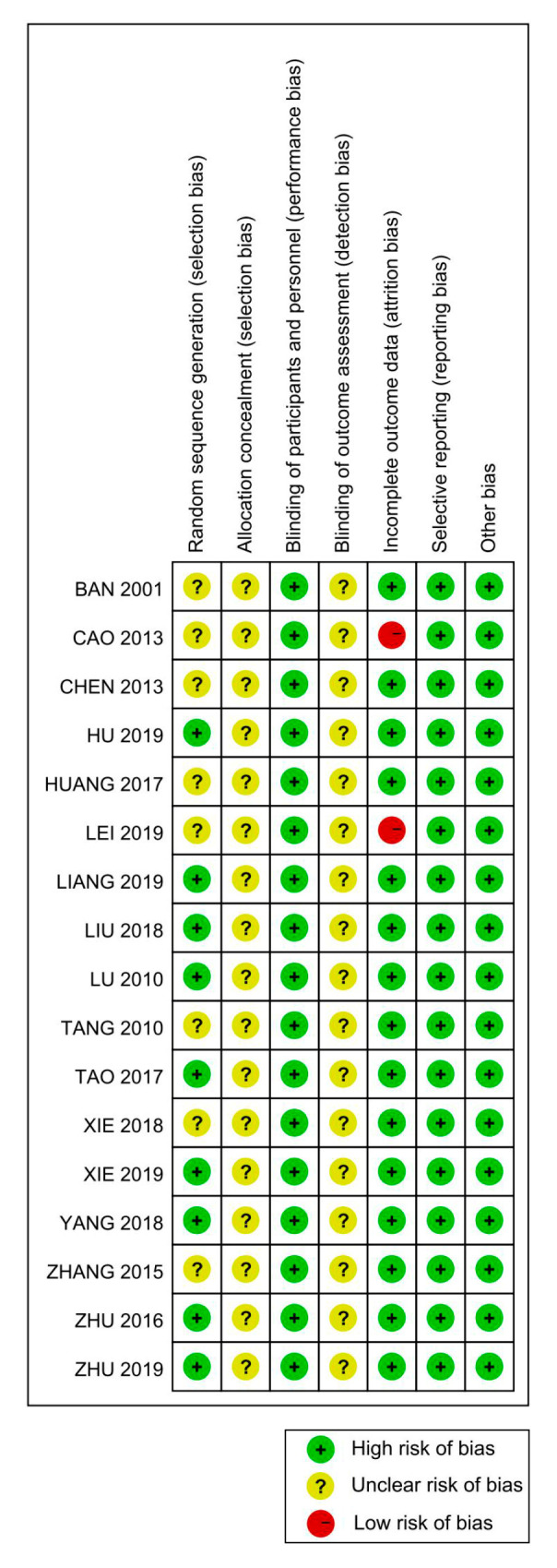
Summary of the risk of bias of included studies.

Figure 2 shows the evaluations of each risk of bias. Allocation concealment and blinding of outcome assessment were evaluated as unclear risks, whereas blinding of participants and personnel was assessed as low risk. For incomplete outcome data, two trials contained instances of participation withdrawal. In general, most studies were evaluated as being of low-risk quality. Six quasi-experimental studies conformed to the JBI critical appraisal checklist. The detailed results are presented in Figure 2 and Figure 3 and Table 2 and Table 3.

### 3.4. Meta-Analysis Results

#### 3.4.1. Symptoms

The data in relation to the total score of the symptoms were collected from 11 RCTs and six quasi-experimental studies using PANSS [10,11,38,39,41,42,43,46,47,49,50], BPRS [12,13,14,37], and SANS [15,40].

**Figure 4:**
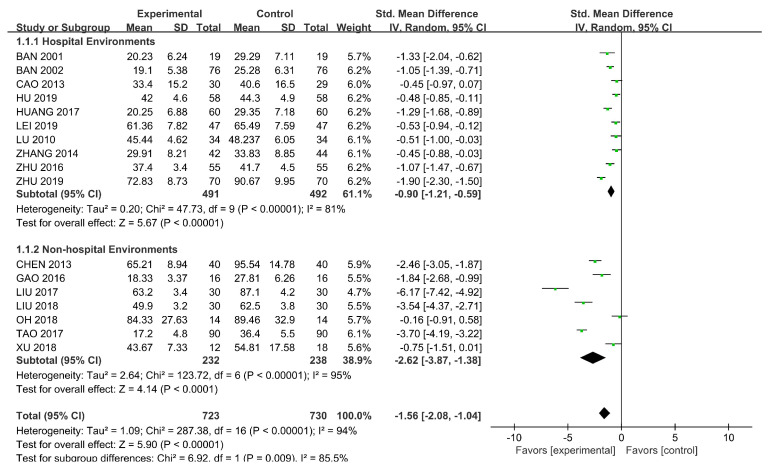
Effects on the symptoms.

SMDs were used because of the different scales. We used a random-effects model (*p* < 0.00001, I^2^ = 94%) and subgroup analysis was conducted according to the intervention settings. The results, as shown in Figure 4, showed a significant difference (SMD = −2.62, 95% CI [−3.87, −1.38], *p* < 0.00001) in the influence of horticultural therapy in non-hospital environments on the total score of symptoms, but the result was less significant when the intervention settings were hospital environments (SMD = −0.90, 95% CI [−1.21, −0.59], *p* < 0.00001). We detected significant differences in the sensitivity analyses when removing Tao (2017) [38] (SMD = −1.39, 95% CI [−1.83, −0.95], *p* = 0.04).

#### 3.4.2. Rehabilitation Outcomes

**Figure 5:**
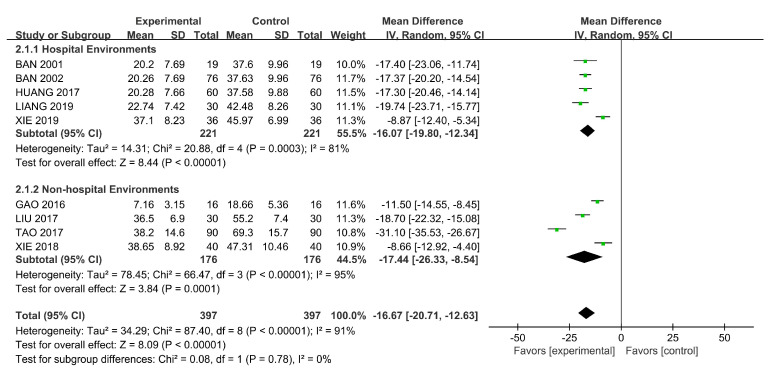
Effects on the rehabilitation outcomes.

The total score of the rehabilitation outcomes was gathered from six RCTs and three quasi-experimental studies using IPROS [12,13,14,37,40,45,47,51,52]. MDs were used because of the uniform standard. We used the random-effects model because of heterogeneity (*p* < 0.00001, I^2^ = 91%) and conducted a subgroup analysis on the basis of the intervention settings. A significantly positive difference was found in the impact of horticultural therapy. We found some differences between the two subgroups (Figure 5). There were significant differences found in the sensitivity analyses when removing Tao (2017) [38] (SMD = −2.01, 95% CI [−2.31, −1.71], *p* = 0.02).

#### 3.4.3. Quality of Life

The total score of the symptoms was determined from three RCTs using SQLS [45] and GQOLI-74 [15,51]. SMD was used because of the different scales. We used the random-effects model because of the existence of heterogeneity (*p* = 0.008, I^2^ = 79%) and conducted a subgroup analysis based on the intervention settings.

**Figure 6:**
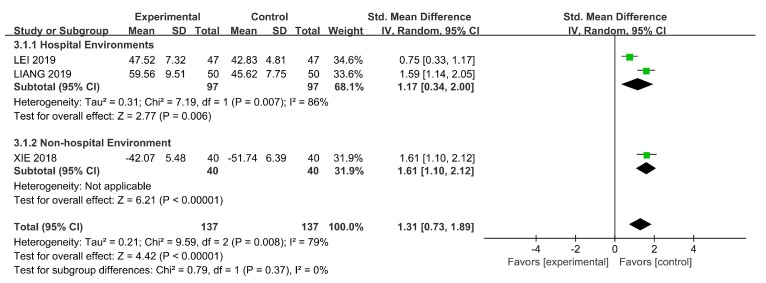
Effects on quality of life.

We found significant differences in the results of horticultural therapy in non-hospital environments on quality of life (SMD = 1.61, 95% CI [1.10, 2.12], *p* = 0.008; Figure 6). When the intervention setting were hospitals, the result was less significant (SMD = 1.17, 95% CI [0.34, 2.00], *p* = 0.007). We detected significant differences in the sensitivity analyses when removing Lei (2019) [15] (SMD = 1.60, 95% CI [1.26, 1.94], *p* = 0.97).

#### 3.4.4. Social Functioning

The total score of social functioning was collected from eight RCTs and a quasi-experimental study using SSPI [36,38,39,45,47,52] and PSP [40,43,48]. SMDs were used because of the different scales. We used the random-effects model because of the existence of heterogeneity (*p* < 0.00001, I^2^ = 98%) and conducted a subgroup analysis considering the intervention settings.

**Figure 7:**
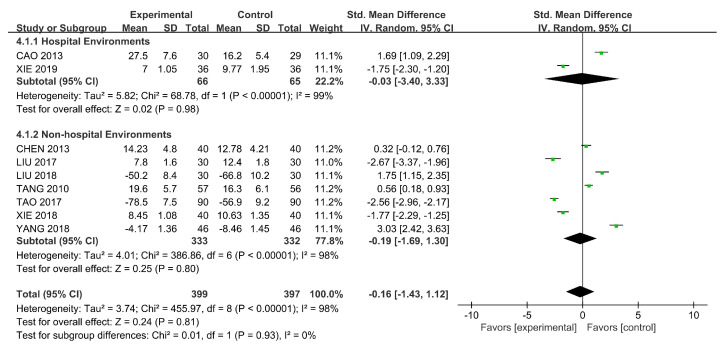
Effects on social functioning.

Figure 7 demonstrates the significant difference (SMD = −0.19, 95% CI [−1.69, 1.30], *p* < 0.00001) in the effect of horticultural therapy in non-hospital environments on the score of social functioning, whereas the result was less significant (SMD = −0.03, 95%CI [−3.40, 3.33], *p* < 0.00001) in hospital settings. We observed some differences between the two subgroups, but no significant difference was found in the heterogeneity analysis when we removed all of the studies one by one.

## 4. Discussion

### 4.1. Outcomes and Processes of Horticultural Therapy

This study focused on the outcomes and processes of horticultural therapy. The findings support the positive effect of horticultural therapy on schizophrenic patients’ symptoms, rehabilitation outcomes, quality of life, and social functioning, as demonstrated by the significant difference in the scores of the experimental and control groups. This shows that horticultural therapy positively impacts the treatment of schizophrenic patients, but the effects vary in different settings (hospital vs. non-hospital environments).

Horticultural therapy can improve the symptoms of schizophrenia by significantly reducing anxiety, depression, stress, and interpersonal sensitivity [53]. To alleviate symptoms such as delusions and hallucinations [54], horticultural activities promote contact between schizophrenic patients and real life.

In terms of rehabilitation outcomes, patients enjoy the natural environment and have more connection with nature, increasing their sensitivity to plants and nature, generating more positive emotions, and promoting their emotional management ability [12,17].

The results also support a positive effect on quality of life. Horticultural activities can help arouse patients’ interest in participating in activities, thus effectively stimulating interest in life [13].

In addition to improving quality of life, this study also clarified the effect of horticultural therapy on social functioning. The research showed that cognitive behavioral therapy (CBT) can improve the social cognition, self-efficacy, and social ability of patients with chronic schizophrenia [18]. Horticultural therapy can be used with CBT to strengthen the sense of accomplishment, responsibility, and belonging [19].

Previous studies focused on the subgroup analysis of the characteristics of activities and populations [23,24,25,26], not on the environment. This study fills this gap and demonstrates that non-hospital environments have a better therapeutic effect on all indicators than hospital environments. The reasons for this result are as follows: (1) there is less chance of a natural experience in hospital environments, whereas non-hospital environments (e.g., farms) immerse people in the sense of beauty and selflessness. Non-hospital environments also have better microclimates, which are beneficial to the healing process, implying a better therapeutic effect. This finding is also consistent with those of some previous studies [55,56,57,58,59,60,61,62,63,64] that greenspace may have a more pronounced effect on individuals with mental illness [65]. A comfortable environment also increases patients’ motivation to participate in activities to reap physical benefits. (2) The types of horticultural therapy activities in hospitals are limited and mainly focus on planting flowers and vegetables and making bonsai; in non-hospital settings, patients can participate in a larger number of activities, such as cultivating plants and picking fruits. More specifically, patients can fully experience the whole growing process throughout the year in non-hospital environments: fertilizing, sowing, watering, weeding, planting, and harvesting. (3) The duration of activities in hospitals was shown to be three (six studies), six (four studies), and 12 months (three studies), whereas the activities in non-hospital settings tended to have a longer follow-up: 6 (five studies), 10 (one study), 12 (one study), and 24 (one study) months. The intervention duration in non-hospital settings was found to be 4–16 h per week, whereas that in hospital settings ranged from 0.5 to 10 h per week. Overall, the treatment duration in most non-hospital environments was longer than in hospital environments, which could also have produced differences in results.

### 4.2. Contributions and Limitations of the Study

The main contributions of this study are as follows. First, this study provides valid evidence supporting the positive effect of horticultural therapy. Our results support a promising avenue of research with relevant application implications. Schizophrenia caregivers (including hospitals and rehabilitation facilities) should provide patients with as many opportunities as possible to participate in horticultural therapy. Therefore, horticultural therapy should be considered an essential tool to treat schizophrenia in future adjuvant therapies for schizophrenic patients. Second, we discussed the differences in the treatment effects in two different environments. We found that non-hospital settings have better healing outcomes, guiding future design and activity organization. The establishment of more professional healing farms or landscapes could be considered to improve the effectiveness of complementary horticultural therapies.

Designing landscapes for horticultural therapy in psychiatric hospitals can make horticultural therapy activities a commonly accepted treatment for patients. The process and the outcome of therapy can provide a further evidence-based reference for future design. We conducted a meta-analysis of horticultural therapy in the auxiliary treatment of schizophrenia. From the analysis, the conclusions provide a basis for evidence-based design to help create a new medical environment based on scientific research data. Thus, patients could receive more optimized treatment, and medical staff could maximize their efficiency and relieve stress in these environments. Evidence-based designs provide a theoretical and empirical foundation for the renovation of the hospital environment and provide a method to promote horticultural therapy.

This study has several limitations. First, the intervention settings were hospitals, agricultural rehabilitation training institutions, farms, and communities. Given the wide range of environments, we only classified these environments into hospital and non-hospital settings instead of more specific environmental subgroups. Second, the studies could be divided into subgroups according to different types of activities to explore which activities are more useful for the recovery of patients with schizophrenia in a future study.

## 5. Conclusions

This meta-analysis showed that horticultural therapy yields positive outcomes in terms of symptoms, rehabilitation outcomes, quality of life, and social functioning of schizophrenic patients. In terms of the environment, different settings can influence treatment; non-hospital environments were shown to have a better therapeutic effect. The result herein can provide a basis and guidance for the future evidence-based landscape design of the treatment of schizophrenia.

Further high-quality studies are needed to explore the substantial therapeutic effect of horticultural therapy. Additional studies on horticultural therapy need to explore more details about the intensity of horticultural therapy activities and the characteristics of the settings in which the activities occur. More research from other countries on horticultural therapy and schizophrenia is needed to contribute to the generalizability of these results.

## Figures and Tables

**Table 1:** Description of the inclusion/exclusion criteria according to population, intervention, comparison, outcomes, and study design (PICOS).

Search Strategy	Details
Inclusion criteria	P: People with schizophrenia
	I: Horticultural therapy
	C: Medication and conventional workshop training
	O: Symptoms, rehabilitation outcomes, quality of life, and social functioning
	S: Randomized controlled trials (RCTs) and quasi-experimental studies
Exclusion criteria	S: Non-original papers (opinion papers, review articles, commentaries, letters, protocols, and reports without quantitative data)
Language filter	English or Chinese
Time filter	From January 2000 to December 2020
Database	PubMed, Cochrane Library, Embase, Science Direct, and China National Knowledge Infrastructure

**Table 2:** Main characteristics of the selected studies.

Author (Publication Year)	Country	Settings	Diagnostic Criteria	Subject			Intervention					Measurement and Outcomes
				Participants E/C	Age E/C	Male (%)	Performer of Intervention	Intervention Duration	Intervention-E	Intervention-C	Follow-Up (Months)	
Ban (2001) [12]	China	Hospital	CCMD-2-R	19/19	25–51	63.16	Gardeners and nurses	60 min per time/five times per week/12 weeks	Horticultural therapy: Planting flowers and making bonsai	Usual schizophrenia care: Medication and conventional work and entertainment treatment	3	BPRS and IPROS
Huang (2017) [13]	China	Hospital	Not mentioned	60/60	60–81	53.33	Gardeners and nurses	Not mentioned	Horticultural therapy: planting flowers	Usual schizophrenia care: medication and conventional work and entertainment treatment	3	BPRS and IPROS
Gao et al. (2016) [14]	China	The agricultural rehabilitation training institution	ICD-10	16/16	55.6 ± 2.3/56.3 ± 2.3	40.63	Horticultural therapists	60 min per time/four times/eight weeks	Horticultural therapy: Seed planting, plant appreciation, cutting propagation, flower pot planting, and taste grown vegetables	Usual schizophrenia care: Rehabilitation training, sanitation, self-care training and medication training	2	BPRS and IPROS
Tang et al. (2010) [36]	China	The agricultural rehabilitation training institution	ICD-10	57/56 36/36	29–64	65.41	Therapeutic specialists	120–180 min per time/five times per week/40 weeks	Horticultural therapy: Planting vegetables, flowers, and fruits	Usual schizophrenia care: Watching TV, listening to music, singing, reading books, playing chess, playing cards, playing table tennis, and cleaning the room	10	SSPI
Ban (2002) [37]	China	Hospital	CCMD-2-R	76/76	40.0 ± 7.96/38.13 ± 9.24	63.16	Gardeners and occupational staff	>300 min a week/12 weeks	Horticultural therapy: Planting flowers and making bonsai	Usual schizophrenia care: Medication	3	BPRS and IPROS
Cao and Wu (2013) [38]	China	Hospital	ICD-10	30/30	42.4 ± 9.3/43.7 ± 9.0	72.88	Three agronomy therapists	90 min per time/once per week/24 weeks	Horticultural therapy: Planting corn	Usual schizophrenia care: Medication	6	PANSS and SSPI
Chen and Jia (2013) [39]	China	The agricultural rehabilitation training institution	ICD-10	40/40	43.26 ± 10.26/45.21 ± 9.87	67.50	Agricultural specialists	8–12 h per week/96 weeks	Horticultural therapy	Usual schizophrenia care: Watching TV, listening to music, singing, reading books, playing chess, playing cards, playing table tennis, and cleaning the room	24	PANSS and SSPI
Tao and Sun (2017) [40]	China	The agricultural rehabilitation training institution	ICD-10	90/90	41.5 ± 6.8/40.4 ± 7.5	Not mentioned	Agricultural specialists, doctors, and nurses	60–120 min per time/5–8 times per week/24 weeks	Horticultural therapy: Planting vegetables and fruits	Usual schizophrenia care: Medication and conventional work and entertainment treatment	6	SANS, IPROS, and PSP
Oh et al. (2018) [10]	Korea	Farm	ICD-10	14/14	42.1 ± 13.0/33.4 ± 9.4	71.43	Two horticultural therapists and one volunteer	120 min per week/10 weeks	Horticultural therapy: Plant cultivating activities	Usual schizophrenia care: Medication, leisure activities, and exercise program	2.5	PANSS and BPRS
Zhu et al. (2016) [11]	China	Hospital	ICD-10	55/55	48.2	43.64	Two rehabilitative therapists	90 min per time/three times per week/12 weeks	Horticultural therapy: Seeding, watering, fertilizing, weeding, and catching pests	Usual schizophrenia care: Medication	3	PANSS
Hu et al. (2019) [41]	China	Hospital	ICD-10	58/58	45 ± 8/48 ± 7	64.66	Horticultural specialists, doctors, and nurses	60 min per session/twice a week/12 weeks	Horticultural therapy: Planting and making garden micro-landscape	Usual schizophrenia care	3	PANSS
Zhu and Zhang (2019) [42]	China	Hospital	ICD-10	70/70	46.97 ± 11.48/46.96 ± 9.54	Not mentioned	Therapeutic specialists, doctors, nurses, and agricultural specialists	60–90 min per time/5–7 h a week/24 weeks	Horticultural therapy: Planting vegetables and raising animals	Usual schizophrenia care	6	PANSS
Lei et al. (2019) [15]	China	Hospital	ICD-10	47/47	36.04 ± 9.52/35.45 ± 7.91	55.32	Agricultural specialists and nurses	60 min per time/once every two days/48 weeks	Horticultural therapy: Turning the ground, sowing, watering, and fertilizing, removing insects, weeding, picking vegetables	Usual schizophrenia care: Medication and conventional work and entertainment treatment, such as music therapy and physical training, group games	12	SANS and GQOLI-74
Liu (2018) [43]	China	The agricultural rehabilitation training institution	ICD-10	30/30	41.4 ± 11.6/40.9 ± 11.3	75.00	Staff	More than 60 min per time/5–8 h per week/24 weeks	Horticultural therapy	Usual schizophrenia care: Medication	6	PANSS and PSP
Yang et al. [44]	China	The agricultural rehabilitation training institution	DSM-IV-TR	46/46	37.72 ± 6.16/38.44 ± 6.76	Not mentioned	Agricultural specialists and staff	60 min per time/seven times per week/24 weeks	Horticultural therapy: Watering, weeding, sowing vegetables, and fertilizing	Usual schizophrenia care: Medication	6	SDSS
Xie (2018) [45]	China	The agricultural rehabilitation training institution	ICD-10	40/40	44.89 ± 4.96/45.03 ± 4.82	56.25	Therapeutic specialists	120 min per time/24 weeks	Horticultural therapy: Planting, pulling weeds, hoeing, watering, and picking fruits	Usual schizophrenia care: Medication and rehabilitation knowledge training, life and social skills training, psychotherapy	6	SSPI, SQLS, and IPROS
Xu et al. (2018) [46]	China	Community	ICD-10	12/16	44.33 ± 9.71/44.19 ± 8.12	Not mentioned	Psychiatrists, nurses, psychological counselors, public health physicians, rehabilitation specialists, social workers, disabled workers with agricultural skills, and family members	More than 60 min per time/twice per week/24 weeks	Horticultural therapy: Turning the ground, sowing, and maintaining and picking vegetables and fruits	Usual schizophrenia care: Medication and conventional work and entertainment treatment	6	PANSS
Liu et al. (2017) [47]	China	The agricultural rehabilitation training institution	ICD-10	30/30	46.4 ± 8.5/46.5 ± 8.2	65.00	Agricultural specialists	More than 60 min per time/5–8 h per week/48 weeks	Horticultural therapy: Fertilizing, sowing, watering, weeding, planting, and harvesting	Usual schizophrenia care: Medication	12	PANSS, IPROS, and SSPI
Zhang et al. (2015) [48]	China	Hospital	DSM-IV	45/38	42.25 ± 9.25/43.26 ± 8.91	100.00	Therapeutic specialists and agricultural specialists	120 min per time/once every two days/24 weeks	Horticultural therapy: Planting, weeding, and fertilizing	Usual schizophrenia care	6	PSP
Zhang et al. (2014) [49]	China	Hospital	ICD-10	42/44	35.42 ± 7.21/38.20 ± 5.41	100.00	Nurses	One hour per day/48 weeks	Horticultural therapy: Breeding, planting vegetables, studying forest and fruit technology, and cultivating flowers	Usual schizophrenia care: Medication	12	PANSS
Lu and Wang (2010) [50]	China	Hospital	ICD-10	34/34	42 ± 12/40 ± 11	61.76	Agricultural specialists	60 min per time/once every two weeks/48 weeks	Horticultural therapy: Planting, weeding, and fertilizing	Usual schizophrenia care: Medication and conventional work and entertainment treatment	12	PANSS
Liang et al. (2019) [51]	China	Hospital	ICD-10	30/30	36.78 ± 8.50/36.73 ± 8.34	66.67	Horticultural therapists	120 min per time/five times per week/12 weeks	Horticultural therapy: Planting	Usual schizophrenia care: medication	3	IPROS and GQOLI-74
Xie and Cao (2019) [52]	China	Hospital	Not mentioned	36/36	45.43 ± 5.14/45.12 ± 5.23	56.94	Agricultural specialists and nurses	Not mentioned	Horticultural therapy:	Usual schizophrenia care: Medication and social function exercise, and psychotherapy	6	SSPI and IPROS

Abbreviations: E, Experimental; C, Control; CCMD, Chinese Classification of Mental Disorders; ICD, International Classification of Diseases; DSM, the Diagnostic and Statistical Manual of Mental Disorders; PANSS, the Positive and Negative Syndrome Scale; BPRS, the Brief Psychiatric Rating Scale; SANS, the Scale for Assessment of Negative Symptoms; IPROS, the Inpatient Psychiatric Rehabilitation Outcomes Scale; SQLS, the Schizophrenia Quality of Life Scale; GQLI-74, the Generic Quality of Life Inventory-74; SSPI, the Scale of Social function in Psychosis Inpatients; PSP, the Personal and Social Performance scale; SDSS, the Social Disability Screening Schedule.

**Table 3:** Risk of bias of the included studies (quasi-experimental studies).

Included Study	1	2	3	4	5	6	7	8	9
Ban (2002) [37]	Yes	Yes	Yes	Yes	Yes	Yes	Yes	Yes	Yes
Gao (2016) [14]	Yes	Yes	Yes	Yes	Yes	Yes	Yes	Yes	Yes
Liu et al. (2017) [47]	Yes	Yes	Yes	Yes	Yes	Yes	Yes	Yes	Yes
Oh et al. (2018) [10]	Yes	Yes	Yes	Yes	Yes	Yes	Yes	Yes	Yes
Xu et al. (2018) [46]	Yes	Yes	Yes	Yes	Yes	Yes	Yes	Yes	Yes
Zhang et al. (2014) [48]	Yes	Yes	Yes	Yes	Yes	Yes	Yes	Yes	Yes

Notes: (1) Is it clear in the study what is the “cause” and what is the “effect” (i.e., there is no confusion about which variable comes first)? Were the participants included in any similar comparisons? (2) Were the participants included in any comparisons similar? (3) Were the participants included in any comparisons receiving similar treatment/care, other than the exposure or intervention of interest? (4) Was there a control group? (5) Were there multiple measurements of the outcome both pre- and post-the intervention/exposure? (6) Was follow-up complete and, if not, were differences between groups in terms of their follow-up adequately described and analyzed? (7) Were the outcomes of participants included in any comparisons measured in the same way? (8) Were outcomes measured reliably? (9) Was an appropriate statistical analysis used?
